# Incomplete functional recovery after delirium in elderly people: a prospective cohort study

**DOI:** 10.1186/1471-2318-5-5

**Published:** 2005-03-17

**Authors:** Melissa K Andrew, Susan H Freter, Kenneth Rockwood

**Affiliations:** 1 Division of Geriatric Medicine, Dalhousie University, Halifax, Nova Scotia, Canada

## Abstract

**Background:**

Delirium often has a poor outcome, but why some people have incomplete recovery is not well understood. Our objective was to identify factors associated with short-term (by discharge) and long-term (by 6 month) incomplete recovery of function following delirium.

**Methods:**

In a prospective cohort study of elderly patients with delirium seen by geriatric medicine services, function was assessed at baseline, at hospital discharge and at six months.

**Results:**

Of 77 patients, vital and functional status at 6 months was known for 71, of whom 21 (30%) had died. Incomplete functional recovery, defined as ≥10 point decline in the Barthel Index, compared to pre-morbid status, was present in 27 (54%) of the 50 survivors. Factors associated with death or loss of function at hospital discharge were frailty, absence of agitation (hypoactive delirium), a cardiac cause and poor recognition of delirium by the treating service. Frailty, causes other than medications, and poor recognition of delirium by the treating service were associated with death or poor functional recovery at 6 months.

**Conclusion:**

Pre-existing frailty, cardiac cause of delirium, and poor early recognition by treating physicians are associated with worse outcomes. Many physicians view the adverse outcomes of delirium as intractable. While in some measure this might be true, more skilled care is a potential remedy within their grasp.

## Background

Delirium is a common presentation of illness in frail older adults and is associated with poor outcomes [1-7]. Risk factors for the development of delirium have been investigated [4,8], but factors that predict poor recovery from delirium remain incompletely understood [9]. A recent study of elderly patients admitted to long-term care facilities found that longer duration of delirium was associated with worse functional outcomes [10]. There is also some evidence that hypoactive delirium is associated with poor outcomes [11-13], but results have been conflicting [14,15]. Additionally, delirium is often under-recognized [16,17], and this non-recognition is not without consequence. Compared with patients whose delirium is detected, patients in whom delirium goes unrecognized by treating doctors and nurses have higher 6-month mortality [18]. Risk factors for under-recognition by nurses include hypoactive delirium, age >=80, vision impairment, and dementia [19]. On the other hand, a recent controlled trial found no benefit of an intervention to increase detection [20], suggesting that better detection without better than our current management might well be inadequate. Still, a single study should neither oblige us to consider that the case is closed, nor deter us from seeking better management methods [21]. Identification of factors predictive of outcome remains important, for prognostication, to advise about future care, and for targeting of better intervention and management strategies.

This study set out to identify factors associated with a composite outcome of incomplete functional recovery or death following an episode of delirium coinciding with an acute-care hospital admission. We evaluated both short term (by hospital discharge) and longer-term (at 6 months post-discharge) outcomes.

## Methods

The study was conducted in tertiary care medical, surgical, and geriatric ward settings in Halifax, Nova Scotia. The cohort was assembled through usual care and follow-up was done as a combination of usual care and research study protocol. Data were collected prospectively on consecutive patients who were diagnosed with delirium according to standard DSM-IIIR criteria by an attending geriatrician as part of usual care. Included patients were admitted to a geriatric service (N = 37) or seen in consultation on an internal medicine (N = 25) or surgical (N = 15) ward. Age, sex, Mini-Mental State Examination (MMSE) [22] at the time of assessment, presence and severity of dementia, pre-morbid function as indicated by the Barthel Index [23], frailty as measured by the Geriatric Status Score (GSS) [24], probable cause of delirium (categorized as medications, infection, cardiac, metabolic, or other/combination), whether delirium had been recognized as such by the treating service according to notes in the medical record, and presence or absence of agitation while delirious were recorded at the time of assessment. All data were obtained from the best available sources, including chart notes, health care professional or family report, and clinical examination. Pre-morbid function and cognitive status were determined based on a combination of the medical records and proxy report. Vital status and functional status were obtained at hospital discharge and 6 months following discharge, in clinic or telephone interviews by a study nurse.

### Measures

The Geriatric Status Score was developed at this institution [24], and used as the basis of a frailty score for community populations [25,26]. Briefly, this ordered categorical scale is used to describe function (including cognition, activities of daily living, mobility, and incontinence) and frailty in older adults and has 7 categories: 1 – healthy, no functional deficits, no cognitive impairment, 2 – mild functional impairment, 3 – moderate functional impairment, 4 – moderate-severe functional impairment, 5 – severe functional impairment, 6 – total dependence, and 7 – terminally ill and expected to die within 30 days. The version of the Barthel Index [27] used here is that modified by Granger, which is scored on a scale of 0 to 100 [23] with 100 indicting independence in mobility and Activities of Daily Living. Underlying dementia was diagnosed using DSM-IIIR criteria [28] and categorized as absent, cognitive impairment no dementia (CIND) [29], mild, moderate, or severe. Most probable cause of delirium was determined using available source including patient chart and patient and family interview. Two criteria were used: laboratory and/or radiologic evidence of the putative cause and temporal association. Probable cause was analyzed in two ways: as a categorical variable grouped according to category of cause (medication/alcohol, infection, cardiac, metabolic, other) and as a binary variable medications/alcohol *vs. *all other causes. This was done to test the hypothesis that delirium caused by medications may confer a better prognosis than other causes [30]. The determination of whether delirium had been recognized was made by one of the investigators (KR) or by a study nurse, using a protocol developed for another study [31]. Briefly, from the written record, we recorded whether any physician responsible for day-to-day care (attending or housestaff) had recorded within 24 hours of the delirium being present the terms "delirium", "acute confusion" or a reasonable synonym. This determination was done at the time of initial assessment by study personnel. Duration of delirium was recorded using best available information, as described in an earlier study [32].

Follow-up information on function was obtained for participants who were alive and contactable, either as part of usual care (n = 29) or in proxy-verified telephone follow-up (n = 21) for this study. The primary outcome was a composite of death or functional decline in both short- (at hospital discharge) and long- (6-months post-discharge) term. Functional decline was defined as a decrease by ≥10 points compared with pre-morbid BI score. This cut-off was chosen to represent clinically detectable functional decline (*e.g*. associated with loss of full independence in one functional domain, or lesser decreases in two separate functional areas). Based on our experience [31,32], this would be clinically detectable (translating into an effect size of ~0.5, given a pooled standard deviation of ~20). Blinding was not possible in usual care follow-up patients.

### Statistical analysis

Data were analyzed using Stata 8 [33] and Statistix 8 [34] analytical software packages. Following descriptive analysis of associations using chi-square testing, logistic regression models were constructed. Functional decline (defined as a decrease of >9 points on the Barthel index) and a composite outcome of death or functional decline were studied at hospital discharge and 6 month follow-up. All models adjusted for age, sex, and frailty according to the GSS.

### Ethics

The project was approved by the Research and Ethics committee of Camp Hill Medical Centre, Halifax, Nova Scotia, Canada. Additional written, informed consent or telephone consent was obtained for the follow-up interview.

## Results

**Figure 1:**
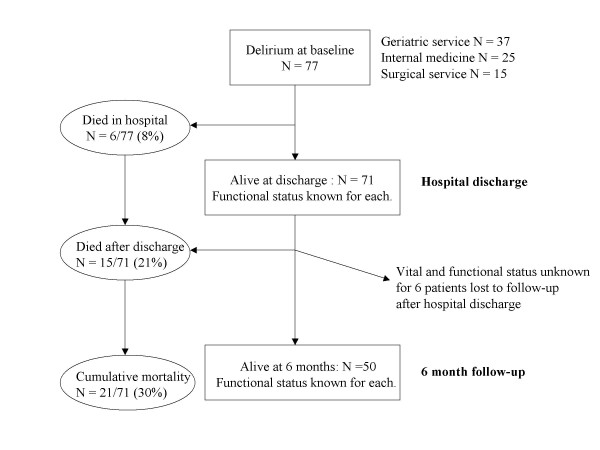
Study cohort flowchart.

Of 77 patients with delirium during their acute-care admission, 6 died in hospital. Vital status at 6 months is known for 65 of the 71 survivors, of whom another 15 had died (6 months mortality = 30%). See Figure 1. At baseline, most patients were frail, with 19 (27%) having a pre-morbid BI score of 100, although a minority had dementia (Table 1). No patient had a pre-morbid BI of zero (lowest score = 42), so all patients had the potential to show a decline in function.

**Table 1:** Characteristics of the study population of patients with delirium (N = 77).

**Patient characteristics**		**Value**	**N**
Age (years)	mean (SD)	78.5 (7.2)	77
	range	64–93 years	
Sex – male	N (%)	34 (44%)	77
Duration of admission	mean days (SD)	38.6 (47.8)	77
	median (interquartile range)	24 (15–41)	
	range	1–292	
Duration of Delirium	mean days (SD)	6.3 (6.1)	77
	median (interquartile range)	5 (2–7)	
	range	1–35	
Medications or alcohol as cause of delirium	N (%)	30 (39%)	76
Cause of delirium	N (%)		76
Medications, alcohol		30 (39%)	
Infection		8 (11%)	
Cardiac		12 (16%)	
Metabolic		2 (3%)	
Other		24 (32%)	
Agitation present	N (%)	12 (18%)	65
Poor recognition of delirium	N (%)	25 (32%)	77
GSS – Frailty	N (%)		77
1 – healthy, independent		4 (5%)	
2 – mild impairment		10 (13%)	
3 – moderate impair.		26 (34%)	
4 – moderate-severe		25 (33%)	
5 – severe impairment		10 (13%)	
6 – totally dependent		2 (3%)	
7 – terminally ill		0	
Dementia			77
None		44 (57%)	
Mild dementia		14 (18%)	
Moderate dementia		15 (19%)	
Severe dementia		4 (5%)	
MMSE score/30 (SD) (on initial assessment)	range 0–27	12.5 (7.4)	75
Barthel Index score (SD)	range		
Pre-morbid	42–100	86.6 (17.4)	77
At hospital discharge	0–100	78.9 (24.9)	71
At 6 month follow-up	6–100	78.2 (22.3)	50
Mean change in Barthel Index (SD)			
Pre-morbid to discharge		-8.9 (19.7)	71
Pre-morbid to 6 month		-12.7 (16.9)	50
Functional decline	N (%)		
At hospital discharge		26 (37%)	70
At 6 months		27 (54%)	50

GSS = Geriatric severity score [21]. MMSE = Mini-Mental State Examination. SD = standard deviation.

Functional status was known for 71 patients at hospital discharge and 50 survivors at 6 months. 6 patients were lost to follow-up after hospital discharge. By 6 months, most patients (48/71 = 68%) had a poor outcome (death or functional decline). Presence and severity of dementia, length of hospital stay, and duration of delirium did not show a statistically significant association with short- or long-term outcomes. Cardiac cause of delirium was associated with 8 times the odds of poor outcome at hospital discharge (95% CI 1.3–47.6) and a trend (p = 0.06) towards poor outcome at 6 months. Causes other than medications were also associated with death or functional decline at 6 months.

Poor recognition of delirium by the primary service was associated with death or poor functional recovery at hospital discharge (p < 0.0001). This is a crude estimate only, as a model that adjusting for age, sex, and frailty was not possible because lack of recognition perfectly predicted poor outcome (*i.e. *no patient who was poorly recognized had a good outcome at hospital discharge). At 6 months, this crude association persisted (p < 0.0001), and was robust to adjustment for age, sex, and frailty (Table 2). 1 patient (4%) whose delirium had been poorly recognized had a good outcome at 6 months. Of those whose delirium was well recognized early in their clinical course, 7 patients (13%) had a poor outcome at hospital discharge, and 25 (53%) had a poor outcome at 6 months.

**Table 2:** Odds of death or functional decline, adjusting for age, sex, and frailty.

	**At hospital discharge** **N = 77**		**At 6 month follow-up** **N = 71**	
	
**Model includes**	**p value **	**OR (95% CI)**	**p value**	**OR (95% CI)**
Age	0.7	1.0 (0.9, 1.1)	0.2	1.0 (1.0, 1.1)
sex	0.4	1.5 (0.5, 4.4)	0.9	1.1 (0.3, 3.4)
Frailty (GSS)	0.008	2.0 (1.2, 3.5)	0.03	1.8 (1.1, 3.2)
Length of admission	0.8	1.0 (1.0, 1.0)	0.5	1.0 (1.0, 1.0)
Duration of delirium	0.2	1.1 (1.0, 1.2)	0.08	1.2 (1.0, 1.4)
BI admission	0.8	1.0 (1.0, 1.0)	0.9	1.0 (0.9, 1.0)
Meds causing delirium	0.1	0.4 (0.1, 1.2)	0.02	0.3 (0.1, 0.8)
Cause: Meds	Baseline		Baseline	
Infection	0.9	0.9 (0.2, 5.8)	0.4	2.2 (0.3, 15.1)
Cardiac	0.02	8.0 (1.3, 47.6)	0.06	9.2 (0.9, 91.3)
Metabolic	1.0	0.9 (0.0, 19.6)	0.08	3.3 (0.9, 12.5)
Other/combo	0.2	2.4 (0.7, 8.5)	**	**
Poor recognition	**	**	0.008	18.2 (2.2, 153.2)
Agitation	0.02	0.1 (0.0, 0.6)	0.2	0.4 (0.1, 1.8)
MMSE when assessed	0.1	0.9 (0.9, 1.0)	0.2	0.9 (0.8, 1.0)
Dementia severity	0.9	1.0 (0.6, 1.7)	0.9	1.0 (0.5, 2.0)
Dementia: none	Baseline		Baseline	
Mild	0.2	0.4 (0.1, 1.8)	0.6	0.7 (0.2, 3.0)
Moderate	0.8	0.8 (0.2, 3.1)	0.7	0.8 (0.2, 3.5)
Severe	0.7	1.6 (0.1, 20.5)	**	**
Change in BI: pre-morbid to discharge			0.07	0.9 (0.9, 1.0)
BI at hospital discharge			0.1	1.0 (0.9, 1.0)

** unable to construct model as success is predicted perfectly

CI = confidence interval, OR = odds ratio, GSS = Geriatric severity score [21], MMSE = Mini-Mental State Examination, BI = Barthel Index.

Adjusting for the effects of age and sex, increasing frailty was associated with poor functional recovery at the time of hospital discharge and at follow-up. Taking age, sex, and frailty into account, agitated delirium appeared to be protective against poor outcome at hospital discharge, but no statistically significant association was seen at 6-month follow-up.

The possibility of interaction was investigated where considered plausible. No evidence of interaction was found between frailty & age, dementia & age, or MMSE score & Barthel Index. Interaction between agitation and poor recognition could not be tested because poor recognition predicted failure perfectly.

## Discussion

Both frailty and poor recognition of delirium by the primary managing service were associated with greatly increased odds of poor outcome, defined as death or functional decline, in both short and long term. Absence of agitation was associated with poor outcome at hospital discharge. We also identified a trend towards a decline in functional status over the course of the hospital admission being a predictor of poor outcome at 6 months. In contrast to the results of another study that found longer duration of delirium symptoms to be associated with poor functional outcomes among patients admitted to long-term care facilities [10], neither duration of delirium nor length of hospital stay was associated with functional recovery in our hospital-based study.

Our findings must be interpreted with caution. Our small sample size (N = 77) likely resulted in our not detecting statistically significant associations between some of the factors investigated and the outcomes, such as the relationship with dementia, which here was swamped by the association with frailty. At a significance level of 0.05, our study had a power of 0.8 to detect a BI difference of 10 points (corresponding to an effect size of 0.7) between patients whose delirium had been well *vs. *poorly recognized, whereas it was under-powered to detect such a difference in outcomes between patients with no dementia *vs. *those with dementia (power 0.6 to detect a difference of 10 points by hospital discharge and power 0.7 at six months). Additionally, as delirium is a clinical diagnosis, there is always some diagnostic uncertainty. This might have impacted both the diagnosis (and thus inclusion of patients in our study population) as well as the assessment of duration of delirium. On the other hand, assessments were done by an experienced geriatrician using predetermined criteria. The outcome measure was a composite of death and poor functional recovery, as defined by a drop of >=10 points on the BI. This endpoint thus relies on the inter- and intra-rater reliability of the BI, which has been shown to be good [35,36]. Additionally, some of the follow-up data were collected by telephone interview. BI scoring based on telephone interview has been found to be reliable [35,37].

Although delirium is common in frail elderly patients, of whom 10–60% have delirium, an estimated 22–66% of cases go unrecognized [16]. Our findings suggest that poor recognition of delirium in hospitalized patients is a risk factor for mortality and poor functional outcomes. This is consistent with findings from a study of elderly patients discharged home from emergency departments, in which undetected delirium was associated with increased 6-month mortality [18]. Interestingly, since all of our patients were seen at some point by a geriatrics service at which time delirium was recognized and management suggestions made, it appears that detection early in the course of delirium is crucial. However, since the precise duration of delirium prior to its recognition is unknown, it is uncertain how long the delay in recognition must be before it becomes associated with adverse outcomes. It is also possible that differences in implementation of management recommendations between clinical services may have confounded the apparent association between recognition and outcomes. The relationship between poor recognition and adverse outcomes was recognized using a composite outcome as developed for the primary analysis. Considered separately, the relative risk (RR) of death given poor recognition of delirium was 10.4 (95% CI 1.3–84.5) at hospital discharge and 2.6 (95% CI 1.3–5.3) at 6 months. The RR of functional decline was 8.5 (4.0–18.0) at hospital discharge and 2.2 (1.4–3.3) at 6 months. While poor recognition was associated with mortality and functional decline, there was no statistically significant association with institutionalization (OR 2.3, 95% CI: 0.7–7.4). However, only 20 patients were discharged to long-term care facilities, so sample size may have been insufficient to demonstrate association. Lack of recognition was also not associated with duration of delirium or length of hospital stay. Insufficient power may have been an issue in these analyses as well. There was no relationship between cause of delirium and lack of recognition (p = 0.2).

Poor recognition of delirium is relative here – ultimately, each patient was recognized to have had delirium, and some attempt at management, however late in the course, was made. Identification of patients in the course of usual care may have resulted in some profiles of delirious patients being disproportionately excluded (*e.g. *hypoactive delirium or mild cases). Without a systematic assay of delirium in patients who were not referred, we cannot be sure of the effect of this selection bias. Arguably, patients in whom delirium was not recognized, who were never referred for geriatrics consultation, and who were thus excluded from the study, might either have been well enough to go home, or were seen to have done so badly that no attempt at improving outcomes was made.

Six patients were lost to follow-up at 6 months, of whom 5 had been well recognized as being delirious by their treating services. In order to assess the impact of missing follow-up data, we conducted a best/worst case scenario sensitivity analysis, the results of which demonstrated our findings to be robust: if all 6 patients whose outcomes were unknown at 6 months were assumed to have had a poor outcome, the odds ratio of unfavourable composite outcome given poor recognition was 16.1 (95% CI: 2.0–133.4), while if all 6 were assumed to have had a good outcome the OR was 11.6 (95% CI: 2.3–57.7).

In our cohort, agitation was apparently protective for both short- and long-term outcomes. This might have been due to agitation leading to better recognition. Considering agitation as the exposure and recognition as the outcome, those who were agitated were less likely to have poor recognition of their delirium. Adjusting for age, sex, and frailty according to the GSS, the odds ratio for poor recognition in patients with agitation (compared to those without) was 0.09 (95% CI 0.0–0.8; p = 0.03). Among those whose delirium was recognized, there was no statistically significant association between presence of agitation and outcome at hospital discharge (p = 0.4) or after 6 months (p = 0.9).

Better outcomes with agitation is consistent with some previous studies, which found hypoactive delirium to be associated with longer hospital stay, more severe illness, increased pressure ulcers and hospital-acquired infections [11], and longer duration of delirium [12]. However, others have reported equivalent [14] or better [15] outcomes in patients with hypoactive delirium. Although such differences may point towards etiological and pathophysiological differences between clinical subtypes of delirium [13,38,39], it is also possible that the agitation of hyperactive patients leads to better recognition by health care staff and thus to more timely and better management of the delirium and its underlying causes [11]. Our finding that absence of agitation was associated with increased odds of poor recognition by the primary treating service lends support to this hypothesis, given that poor recognition was also associated with much higher odds of poor outcome at both hospital discharge and 6-month follow-up.

Additionally, we found that among patients whose delirium had been appropriately recognized, agitation was not associated with better or worse outcome. This lends support to the idea that agitation is beneficial only in so far as it draws attention to the patient and his or her delirium rather than signaling an intrinsically more benign pathophysiological state.

Adjusting for frailty, age, and sex, cardiac cause of delirium was associated with worse outcomes at hospital discharge, and medications as cause were found to portend a better outcome at 6 months. This is consistent with previous findings [30] and may point towards differences in underlying pathophysiology.

Interestingly, we found that the magnitude of change in BI scores from pre-morbid to hospital discharge, more so than the absolute value of the discharge BI, may be a relevant predictor of death and poor functional recovery 6 months following delirium. This is only a trend (p = 0.07), although a larger sample might have found a statistically significant difference. Of note, it is also consistent with an earlier study, [40] which found that change in the BI was associated with adverse outcomes by the time of hospital discharge. Marked change in function over the course of hospital admission may reflect a more severe delirium associated with greater momentum of a downward spiral from which functional recovery is difficult. Adjusting these models for length of hospital stay did not affect the results.

## Conclusion

We have identified some factors that are associated with unfavourable outcomes at hospital discharge and at 6-month follow-up in patients who have had an episode of delirium. While pre-existing frailty and cause of delirium are not readily modifiable risk factors, poor recognition by treating physicians early in the course of delirium may be, suggesting that a potential remedy for the poor outcomes associated with delirium may be within the grasp of attentive practitioners. While further research is needed, our findings contribute to the literature on the important subject of patients' recovery following delirium. It is our hope that awareness of factors that portend a poor outcome will be useful in discussions relating to management, prognosis and future care needs for older adults with delirium.

## Authors' contributions

KR collected the clinical data. All three authors participated in the design of the analyses. MKA did the analyses and wrote the first draft of the paper. SHF & KR reviewed the analyses and revised the manuscript. All authors read and approved the final manuscript.

## Competing interests

The author(s) declare that they have no competing interests.

## Pre-publication history

The pre-publication history for this paper can be accessed here:


